# Metallothionein–Kidney Bean Polyphenol Complexes Showed Antidiabetic Activity in Type 2 Diabetic Rats by Improving Insulin Resistance and Regulating Gut Microbiota

**DOI:** 10.3390/foods12163139

**Published:** 2023-08-21

**Authors:** Zhaohang Zuo, Weiqiao Pang, Wei Sun, Baoxin Lu, Liang Zou, Dongjie Zhang, Ying Wang

**Affiliations:** 1 College of Food Science, Heilongjiang Bayi Agricultural University, Daqing 163319, China; byndzzh1994@163.com (Z.Z.); hljbypwq@163.com (W.P.); swsw0102@163.com (W.S.); zhangdongjie@byau.edu.cn (D.Z.); 2 Key Laboratory of Coarse Cereal Processing, Ministry of Agriculture and Rural Affairs, Sichuan Engineering & Technology Research Center of Coarse Cereal Industralization, School of Food and Biological Engineering, Chengdu University, Chengdu 610106, China; zouliang@cdu.edu.cn; 3 National Coarse Cereals Engineering Research Center, Daqing 163319, China

**Keywords:** metallothionein, kidney bean polyphenols, type 2 diabetes mellitus, hypoglycemic, gut microbiota

## Abstract

Previous studies have shown that interaction between polyphenols and proteins can benefit health, but the mechanism of its antidiabetic effect has not been thoroughly elucidated. Therefore, this study aimed to investigate the impact of the metallothionein (MT)–kidney bean polyphenol complex on the blood glucose levels and gut microbiota of rats with type 2 diabetes mellitus (T2DM) induced by a high-fat diet combined with streptozotocin (STZ). After 7 weeks of intervention, the MT–kidney bean polyphenol complex can significantly improve the loss of body weight, the increase in blood glucose and blood lipids, and insulin resistance caused by T2DM in rats. In addition, it can effectively alleviate the damage to the pancreas and liver in rats. The MT–kidney bean polyphenol complex also significantly increased the concentrations of six short-chain fatty acids (SCFAs) in the intestinal contents of rats, especially acetic acid, propionic acid, and butyric acid (296.03%, 223.86%, and 148.97%, respectively). More importantly, the MT–kidney bean polyphenol complex can significantly reverse intestinal microflora dysbiosis in rats caused by T2DM, increase intestinal microorganism diversity, improve the abundance of various beneficial bacteria, and reshape the gut microbiota. In summary, the hypoglycemic effect of the MT–kidney bean polyphenol complex and its possible mechanism was expounded in terms of blood glucose level, blood lipid level, and gut microbiota, providing a new perspective on the development of the MT–kidney bean polyphenol complex as functional hypoglycemic food.

## 1. Introduction

Diabetes, as one of the critical problems that threatens human health today, seriously affects people’s quality of life [1]. According to the latest statistics of the International Diabetes Federation (IDF), 10.5% (537 million) of adults (20–79 years old) worldwide had diabetes in 2021, and the number of diabetes cases is expected to rise to 643 million by 2030 [2]. Diabetes comes in two basic varieties, type 1 diabetes mellitus (T1DM) and type 2 diabetes mellitus (T2DM). Currently, the number of patients with T2DM accounts for more than 90% of all diabetic patients [3]. T2DM is seen as a complicated metabolic condition brought on by target organ insulin resistance and pancreatic beta-cell malfunction, which is mainly characterized by hyperglycemia and fat metabolism disorders [4]. T2DM is a chronic and complex disease that requires long-term treatment with various approaches and drugs to prevent or postpone complications and improve patients’ quality of life [5]. This requires the daily management of blood glucose and lipid levels, body weight, cardiovascular risk factors, and complications [6]. However, some drugs used to treat T2DM usually cause several harmful effects, including vitamin B12 deficiency, functional dyspepsia, hepatic injury, and renal injury [7]. Therefore, the development of safe and non-toxic natural products to supplement or replace these drugs to reduce side effects has become a current research focus [8].

The gut microbiota, as a crucial participant in glucolipid metabolism, plays a vital physiological role in host balance and health, and its diversity affects the development of metabolic diseases, including obesity and diabetes [9,10]. Therefore, the gut microbiota has been considered a potential target for dietary intervention or drug therapy in T2DM [11]. One of the possible ways in which the gut microbiota interferes with metabolic diseases is that some beneficial intestinal microorganisms ferment undigested substances to produce short-chain fatty acids (SCFAs), especially acetic acid and butyric acid [12]. By efficiently regulating glucose uptake and use in the liver, skeletal muscle, and adipose tissue of the body, SCFAs can enhance insulin function and blood glucose homeostasis. [13,14]. For example, butyric acid can improve insulin sensitivity, while butyric-acid-producing-bacteria levels in patients with T2DM decrease [15]. Therefore, SCFAs are essential regulators of energy and glucose homeostasis [16].

Metallothionein (MT) is a cysteine-rich (30–33% of total amino acids), low-molecular-weight protein (6–7 kDa) that binds to metals [17]. It was first found by Margoshes and Vallee in horse kidney cells that accumulate cadmium [18]. MT has been confirmed to exist widely in most organs and tissues of microorganisms, and in higher animals, plants, and humans [19]. As a highly effective antioxidant regulator in vivo, MT can effectively participate in the quenching of reactive oxygen species, block the formation chain of active oxygen, and protect the organism from superoxide invasion [20]. The results of Beattie et al. showed that MT had a protective effect in controlling metabolism and regulating energy balance [21]. This provides the possibility that MT interferes with the course and complications of metabolic diseases, including diabetes and obesity.

Several studies have found a link between the consumption of legumes and a lower risk of metabolic disorders such as T2DM, hyperlipemia, and cardiovascular diseases [22,23]. As one of the most important legume crops in the world, the kidney bean (*Phaseolus vulgaris* L.) is rich in protein, essential amino acids, vitamins, dietary fiber, and other nutrients [24]. At the same time, the kidney bean is also a high-quality source of phenolic compounds, so it has high edible and medicinal value, and has great potential in the prevention and treatment of diseases [25]. As a natural hypoglycemic agent, the anti-diabetic effect of plant polyphenols has been widely confirmed in cell, animal, and human clinical trials [26]. Current studies have shown that plant polyphenols and proteins can work together synergistically in terms of antioxidant, anti-inflammatory, and anti-cancer effects, and the regulation of intestinal flora [27,28,29]. However, the potential antidiabetic activity of protein–polyphenol complexes has not been revealed.

Therefore, the goals of this study were to measure the serum biochemical indices, to observe the histopathological sections, and combined with the diversity and abundance of intestinal flora in rats, to analyze the hypoglycemic effect of the MT–kidney bean polyphenol complex on rats with T2DM and its mechanism. This study provides a potential possibility for the MT–kidney bean polyphenol complex in developing antidiabetic functional foods.

## 2. Materials and Methods

### 2.1. Materials

Purple speckled kidney beans (*Phaseolus vulgaris* L.) were provided by Heihe (Heilongjiang, China) (Size: 220–240/100 g. Appearance: a regular shape, no damage.). Metallothionein (MT) was purchased from Shanghai Yuanye Biotechnology Co., Ltd. (Shanghai, China). Streptozotocin (STZ) and metformin were purchased from Sigma (Sigma-Aldrich, St. Louis, MO, USA). High-fat feed (HD001) was provided by Beijing Botai Hongda Biotechnology Co., Ltd. (Beijing, China).

### 2.2. Extraction of Polyphenol from Kidney Beans Using the Ultrasound-Assisted Method

The kidney bean was soaked in purified water at 25 °C for 12 h, then spread evenly in a sterilized bean sprout machine. The water was changed every 12 h and cultivation took place it in a ventilated place for 4–5 days. When the length of the bud was approximately 8–10 cm, the kidney bean was removed and dried (40 °C, 24 h); then, it was crushed, sealed, and stored at −20 °C. The extraction conditions of the polyphenols were as follows: According to the ratio of material to liquid being 1:25, the kidney bean sprout powder was added to the 60% ethanol solution and shaken well. After 20 min, it was treated with 350 W ultrasonic power, and the supernatant was obtained by centrifugation (4000× *g*, 20 min), and concentrated at 45 °C. Purification was carried out using an AB-8 macroporous resin (Cangzhou Baoen Adsorption Material Technology Co., Ltd., Hebei, China), and then the sample was freeze-dried.

The sample extracts were analyzed using a UPLC-QE-Orbitrap MS system (Thermo Fisher Scientific, San Jose, CA, USA). Analytical conditions were as follows: UPLC: column, Waters ACQUITY UPLC HSS T3 (1.8 µm, 2.1 mm × 50 mm); column temperature, 40 °C; flow rate, 0.3 mL/min; injection volume, 2 µL; solvent system, water (0.1% acetic acid): acetonitrile (0.1% acetic acid); gradient program, 90:10 *V*/*V* at 0 min, 90:10 *V*/*V* at 2.0 min, 40:60 *V*/*V* at 6.0 min, 40:60 *V*/*V* at 8.0 min, 90:10 *V*/*V* at 8.1 min, and 90:10 *V*/*V* at 12.0 min. The total phenolic content of the kidney bean polyphenol extracts was (82.92 ± 2.37)%, and the main polyphenolic compounds of the purified kidney bean polyphenol extracts were protocatechuic acid, ferulic acid, and catechin (Supplementary Table S1).

### 2.3. Preparation of the MT–Kidney Bean Polyphenol Complex

The MT–kidney bean polyphenol complex was prepared according to previous studies [30]. The MT sample was dispersed in PBS buffer (0.01 M, pH 7.0) to obtain a 10 mg/mL MT solution. The kidney bean polyphenol sample was dissolved in PBS buffer (0.01 M, pH 7.0) to obtain a solution of 1.0% (*w*/*v*), and the pH was adjusted to 7.0 with a NaOH solution (1 M). The kidney bean polyphenol solution was added to the MT solution; the ratio of MT to kidney bean polyphenol was 1:4 (*v*/*v*) and it was stirred for 2 h (25 °C, 1000 r/min) to form the MT–kidney bean polyphenol complex. The samples obtained were dialyzed at 4 °C for 12 h to remove free polyphenols and then vacuum freeze-dried for later use.

### 2.4. Interaction between MT and Kidney Bean Polyphenol

The fluorescence spectra of the MT–kidney bean polyphenol complexes were measured on an RF-6000 Spectro Fluorophotometer (Shimadzu Co., Ltd., Kyoto, Japan). The excitation wavelength was 350 nm, and the emission wavelength was tuned to range between 350 and 520 nm. The X-ray crystal structure of metallothionein (PDB ID: 4MT2) was obtained from the Protein Data Bank, and molecular docking was conducted using AutoDock Tools 1.5.6 software (The Scripps Research Institute, La Jolla, CA, USA).

### 2.5. Animal and Experimental Design

Forty-eight healthy, specific, pathogen-free (SPF), male Wistar rats (permit number: SCXK (Liao) 2020-0001), aged 5–6 weeks, with an initial weight of 220 ± 20 g, were obtained from Changsheng Biotechnology Co., Ltd. (Benxi, Liaoning, China). The animals were raised in an SPF animal house with 12 h/12 h simulating cyclic light, ambient temperature of (23 ± 2) °C, and relative humidity of (50 ± 10)%. The animal experiments involved were conducted in strict accordance with the laws of the People’s Republic of China and in strict compliance with the international standards of the Institution Animal Care and Use Committee. And, all procedures were examined and filed by the Science and Technology Ethics Committee of Heilongjiang Bayi Agricultural University on 12 May 2022 (approval number: SPXY2022001).

**Figure 1:**
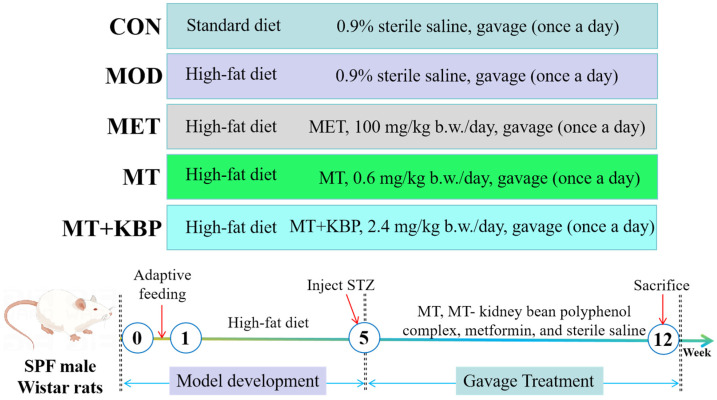
Modeling and in vivo experimental design of rats with T2DM.

After 1 week of adaptive feeding, 8 rats were randomly selected as the normal group (CON) and fed a standard diet (73.5% corn starch, 20% wheat bran, 5% fish meal, 1% farina, and 0.5% sodium salt). The remaining rats were fed a high-fat diet (53.51% corn starch, 14.6% wheat bran, 3.6% fish meal, 0.73% farina, 0.56% sodium salt, 1.2% cholesterol, 5.8% egg yolk powder, 10% sucrose, and 10% lard). After 4 weeks of feeding, rats were fasted without water for 12 h (overnight) and STZ (25 mg/kg body weight) was injected intraperitoneally twice within 72 h; a citric acid buffer of the corresponding quality was injected into the CON group. A total of 72 h after intraperitoneal injection, tail vein blood was taken to determine the value of fasting blood glucose (FBG) ≥ 11.1 mmol/L, accompanied by assessment of dull hair color, slow exercise, and other characteristics. The appearance of these characteristics was considered the successful model of T2DM [31]. The Rats with T2DM were randomly divided into 5 groups (*n* = 8): model group (MOD), metformin group (MET, 100 mg/kg body weight/day), metallothionein group (MT, 0.6 mg/kg body weight/day), and MT–kidney bean polyphenol complex group (MT+KBP, 2.4 mg/kg body weight/day). Based on our previous studies [32], the rat-equivalent dose of MT and the MT–kidney bean polyphenol complex was based on body surface area conversion factors per standard method. Meanwhile, the rats in the CON and MOD groups were given 0.9% sterile saline for 7 weeks (Figure 1).

### 2.6. Sample Collection and Biochemical Analysis

In the experiment, the FBG and body weight of rats were monitored every week after fasting for 12 h. The oral glucose tolerance test (OGTT) was carried out in the 12th week of the experiment. The experimental rats were given a 1 mL glucose solution according to 2 g/kg body weight via intragastric administration. The blood glucose values of rats after administration at 0, 30, 60, and 120 min were measured with a Sannuo blood glucose meter (Sannuo Biosensor Co., Ltd., Changsha, China), and the area under the curve (AUC) was determined according to the following formula [33]:

AUC = (A + B) × 0.25 + (B + C) × 0.25 + (C + D) × 0.5(1)

The formulae represent the blood glucose values of rats after administration at different time points, as follows: Formula A, blood glucose value of rats—0 min; Formula B, blood glucose value of rats—30 min; Formula C, blood glucose value of rats—60 min; Formula D, blood glucose value of rats—120 min.

After 7 weeks of intervention, the rats were anesthetized with pentobarbital sodium (1 mg/kg body weight), and the blood was collected from the abdominal aorta. The serum was centrifuged for 10 min under the conditions of 4 °C and 1800× *g* and separated and stored at −80 °C. The fresh livers and pancreases of all rats were collected and rapidly frozen in liquid nitrogen for further analysis. The livers and pancreases were washed with normal saline, fixed with 4% paraformaldehyde, and used to make pathological sections for observation. The intestinal contents of rats were also collected and stored at −80 °C to determine of SCFAs and gut microbiota. Serum biochemical indices of rats, including insulin, triglyceride (TG), total cholesterol (TC), low-density-lipoprotein cholesterol (LDL-C), high-density-lipoprotein cholesterol (HDL-C), alanine aminotransferase (ALT), and aspartate aminotransferase (AST) were detected by the assay kits (Institute of Bioengineering, Nanjing, China). At the same time, according to the insulin homeostasis model, the homeostasis index of insulin resistance (HOMA-IR) was evaluated and calculated according to the following formula [34]:

HOMA-IR = FBG (mmol/L) × Insulin (mIU/L)/22.5(2)

### 2.7. Histopathological Analysis

The tissues of the pancreas and liver were fixed in paraffin and cut into 5 µm tissue sections. After staining with hematoxylin and eosin (H&E), the sections were observed under the microscope (100×).

### 2.8. Determination of SCFAs

The level of SCFAs was determined by referring to the method of Fang et al. with some modifications [35]. Simply, 25 mg of intestinal content was suspended in 500 µL of water (containing 0.5% phosphoric acid) and frozen for 3 min. The sample was treated with ultrasonic treatment for 10 min (50 Hz), and then centrifuged for 15 min at 13,000× *g* and 4 °C. The supernatant was transferred to a 1.5 mL tube, and 0.2 mL n-butanol solvent (including internal standard 2-ethylbutyric acid, 10 µg/mL) was added. After vortexing for 10 s, the sample was extracted by ultrasound for 10 min. Finally, the sample was centrifuged for 5 min at 13,000× *g* and 4 °C, and the supernatant was stored for follow-up testing. The contents of SCFAs were analyzed by the Agilent 8890B-7000D GC-MS system (Agilent Technologies Inc., Santa Clara, CA, USA) [36].

### 2.9. Analysis of Intestinal Microorganisms by the 16SrRNA Amplification Sequence

The bacterial DNA was extracted from the intestinal contents of rats using a Qubit dsDNA analysis kit (Thermo Fisher Scientific, Waltham, MA, USA) [37]. The amplification primers of 16s rRNA gene (V3-V4 region) were 338F (5′-ACTCCTACGGAGGCAGCAGCAGCAGCAG-3′) and 806R (5′-GGACTACHVGGTWTCTAAT-3′). The construction of a high-throughput sequencing library and Illumina MiSeq sequencing were carried out at Majorbio Biopharmaceutical Technology Co., Ltd. (Shanghai, China).

After that, the original data were spliced and filtered to obtain useful data. The Bayesian RDP Classifier algorithm was used to classify and analyze the operational taxon (OTU), aggregation degree, and object species. The sequences were clustered into the same OTUS based on more than 97% similarity (USEARCH, http://drive5.com/usearch/ version 11, accessed on 20 October 2022). The representative sequence of each OTUS was labeled to obtain the corresponding microbial species information and distribution. Alpha diversity was calculated using Mothur software (version v.1.30.2) (http://www.mothur.org/, accessed on 20 October 2022). The significance of beta diversity was analyzed with QIIME 1.9.1 software (http://qiime.org/install/index.html, accessed on 20 October 2022). Based on the logarithmic transformation of OTUs, the phylogenetic tree was constructed by the neighborhood connection method using Mega software (version 7.0, http://www.megasoftware.net, accessed on 20 October 2022). The heat map of microbial species was drawn using the R programming language.

### 2.10. Statistical Analysis

All experimental data were statistically analyzed using SPSS V20.0 software (IBM Co., Armonk, NY, USA), and the experimental results were expressed by mean ± standard deviation. A one-way analysis of variance (ANOVA) test and Tukey’s HSD test were used. The values with *p* < 0.05 were considered statistically significant.

## 3. Results

### 3.1. Interaction between MT and Kidney Bean Polyphenol

**Figure 2:**
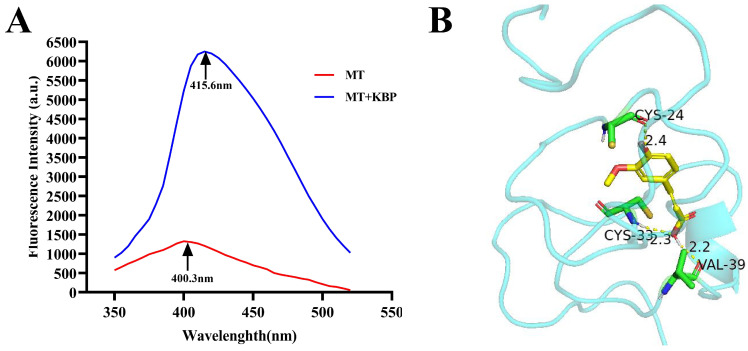
Interaction between MT and kidney bean polyphenol. (**A**) Fluorescence spectra; (**B**) The docking analysis of the binding structure of MT and kidney bean polyphenol.

The change in fluorescence intensity could be used to assess the interaction between proteins and polyphenols [38]. The wavelength of peak luminescence intensity shifted from 400.3 nm of MT to 415.6 nm of MT+KBP (Figure 2A). Metallothionein does not contain aromatic amino acids, so its fluorescence intensity is very low, while MT+KBP samples have an increased fluorescence intensity and a redshift phenomenon, in which metallothionein binds to kidney bean polyphenols on its surface. Additionally, the MT and kidney bean polyphenol molecules formed hydrogen bonds with CYS-24, CYS-33, and VAL-39 residues (hydrogen bond lengths of 2.4, 2.3, and 2.2, respectively) to obtain the stable binding structure of the MT–kidney bean polyphenol complex (Figure 2B).

### 3.2. Effect of the MT–Kidney Bean Polyphenol Complex on the Blood Glucose Level of Rats with T2DM

**Figure 3:**
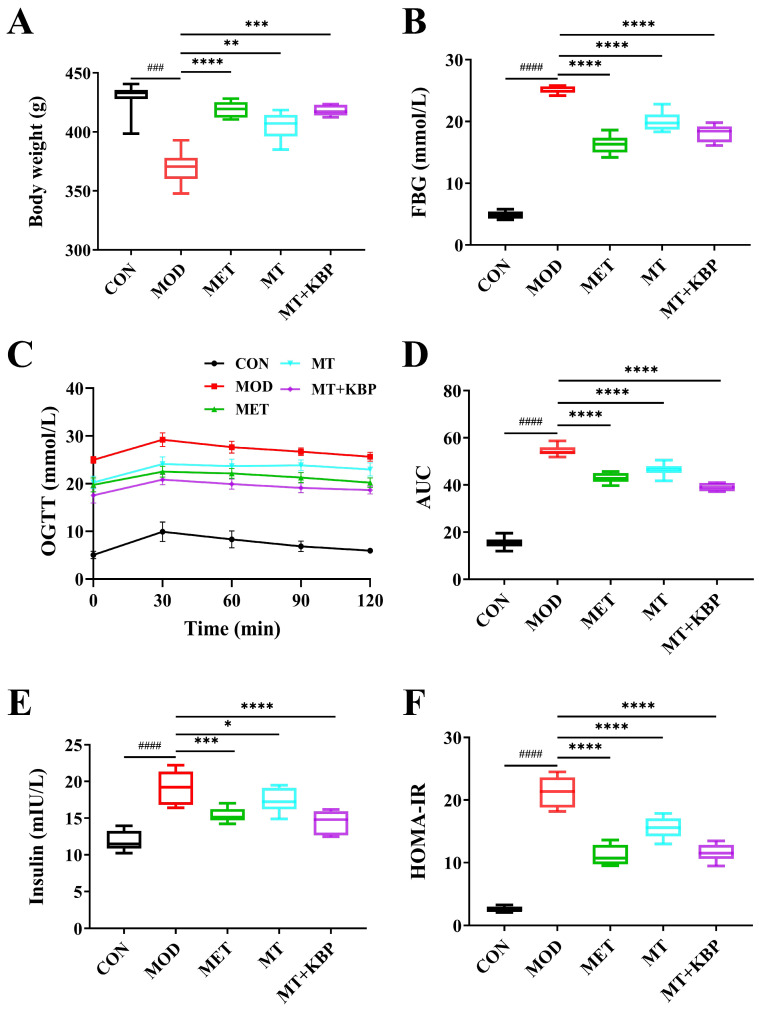
Effect of the MT–kidney bean polyphenol complex on blood glucose level in rats with T2DM. (**A**) Body weight; (**B**) FBG; (**C**) OGTT; (**D**) AUC; (**E**) insulin, INS; (**F**) insulin resistance (HOMA-IR). Data are expressed by mean ± standard deviation, *n* = 8. (### *p* < 0.001 and #### *p* < 0.0001 versus CON; * *p* < 0.05, ** *p* < 0.01, *** *p* < 0.001, and **** *p* < 0.0001 versus MOD).

As is shown in Figure 3A, the body weight of rats in the MOD group was significantly lower than in the CON group (*p* < 0.001). This was consistent with the symptoms of weight loss in patients with diabetes [39]. Compared to the MOD group, the body weight of the rats in the MET group, MT group, and MT+KBP group increased by 13.36% (*p* < 0.0001), 9.79% (*p* < 0.01), and 13.15% (*p* < 0.001), respectively.

In comparison to the CON group, the FBG level was significantly higher than that in the MOD group (*p* < 0.0001) (Figure 3B). Until the end of the experiment (12th week), the FBG level in the MT group and MT+KBP group decreased by 19.97% and 27.86% compared with the MOD group, respectively. And, the MT–kidney bean polyphenol complex had a more apparent hypoglycemic effect than MT alone (*p* < 0.05). Figure 3C,D shows the changes in blood glucose in rats during OGTT. After oral glucose administration for 30 min, the blood glucose level of all experimental rats reached its peak. At each time point, the level of blood glucose in the MOD group was significantly higher than that in the CON group (*p* < 0.0001), suggesting impaired glucose tolerance in rats with T2DM. Compared with the MOD group, the blood glucose level in the MT group and MT+KBP group decreased by 14.77% and 28.18%, respectively, indicating that the intervention effect of the MT–kidney bean polyphenol complex was better than with MT (*p* < 0.05).

The levels of serum insulin and HOMA-IR of rats in each group are shown in Figure 3E,F. Insulin and HOMA-IR levels in the MOD group were significantly higher than those in the CON group (*p* < 0.0001). After 7 weeks of intervention, insulin levels in the MT group and the MT+KBP group were 8.70% and 24.60% lower than in the MOD group, and the HOMA-IR was lower by 27.00% and 45.72% compared to the MOD group, respectively. Among them, the intervention effect of the MT–kidney bean polyphenol complex on insulin level was relatively better (*p* < 0.05).

### 3.3. Effect of the MT–Kidney Bean Polyphenol Complex on Biochemical Indicators in rats with T2DM

**Figure 4:**
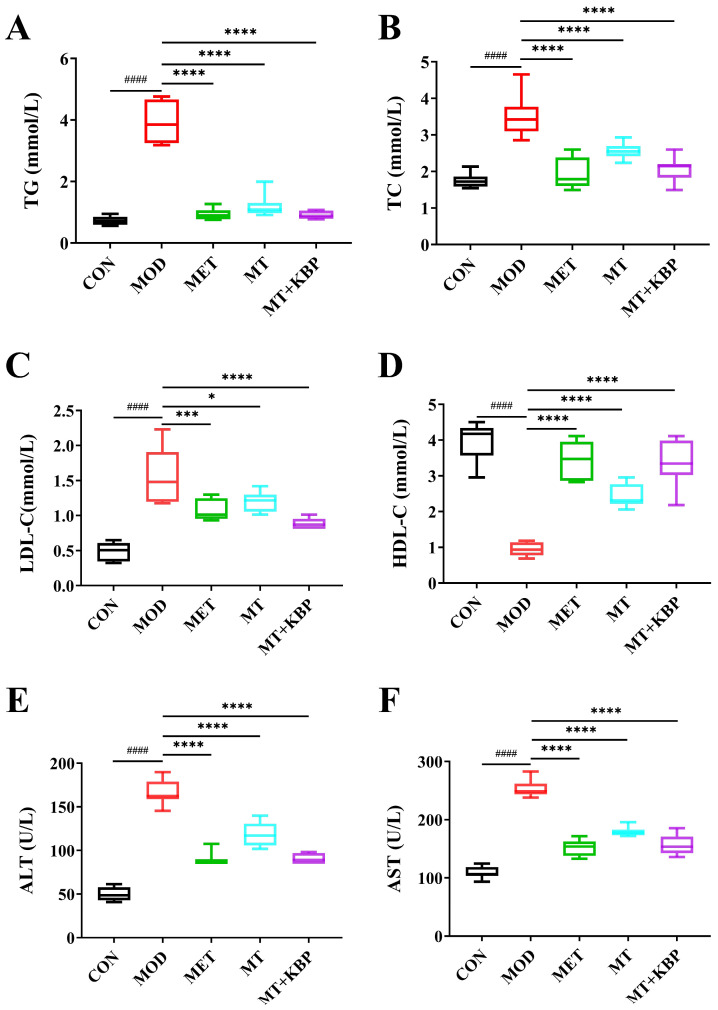
Effect of the MT–kidney bean polyphenol complex on biochemical indicators in rats with T2DM. (**A**) TC; (**B**) TG; (**C**) LDL-C; (**D**) HDL-C; (**E**) ALT; (**F**) AST. Data are expressed by mean ± standard deviation, *n* = 8. (#### *p* < 0.0001 versus CON; * *p* < 0.05, *** *p* < 0.001, and **** *p* < 0.0001 versus MOD).

According to previous research, diabetic patients usually have dyslipidemia. This is mainly characterized by increased levels of TG, TC, and LDL-C, and decreasing levels of HDL-C [40]. The blood lipid level of rats in each group is shown in Figure 4A–D. Compared with the CON group, the levels of TG, TC, and LDL-C in the MOD group were significantly higher, while the level of HDL-C was significantly lower (*p* < 0.0001). This further confirmed that rats with T2DM had symptoms of blood lipid levels. After 7 weeks of intervention, the TG, TC, and LDL-C in the MT group and MT+KBP group decreased, and the MT+KBP group decreased by 76.65%, 40.52%, and 42.95% compared with the MOD group (*p* < 0.0001), respectively. On the other, the HDL-C of the MT group and the MT+KBP group was 159.59% and 256.94% higher than that in the MOD group, respectively. Taking these results together, the intervention effect of the MT–kidney bean polyphenol complex was relatively better (*p* < 0.05).

Compared to the CON group, the ALT and AST levels of rats in the MOD group were significantly higher (*p* < 0.0001) (Figure 4E,F). At the same time, compared with the MOD group, the ALT level decreased by 46.48%, 29.01%, and 46.00%, respectively, while AST decreased by 40.07%, 29.06%, and 38.01%, respectively. And, the intervention effects of the MT–kidney bean polyphenol complex on ALT and AST in rats were better than those of the single MT.

### 3.4. Effects of the MT–Kidney Bean Polyphenol Complex on Histopathology of Pancreas and Liver in Rats with T2DM

**Figure 5:**
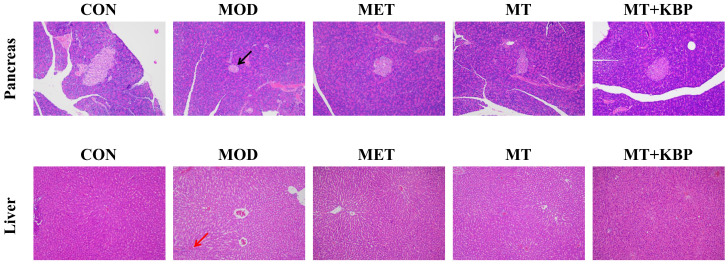
Effects of the MT–kidney bean polyphenol complex on histopathology of the pancreas and liver in rats with T2DM (magnification, 100×). Islet damage is represented by the black arrow, and inflammatory infiltration is represented by the red arrow.

The histological changes in the pancreas in each group are shown in Figure 5. In the CON group, islet cells were normal, intact, and uniformly arranged, and no significant pathological changes, such as the growth and infiltration of inflammatory cells, were found. In the MOD group, the islet structure was atrophied and deformed, and some islet cells showed degeneration, necrosis, vacuolization, or dissolution. After 7 weeks of the experiment, the MET, MT, and MT+KBP groups were able to repair pancreatic tissue injury to varying degrees. Among them, the repair effect of the MT–kidney bean polyphenol complex was the most significant: the morphology of islet cells tended to be complete, the cell edge was clear, and the infiltration and proliferation of inflammatory cells improved.

The pathological observation of the liver tissue of the rats in each group can also be seen in Figure 5. In the CON group, the hepatocytes were normal and arranged in order, the central vein cells were intact, there were no clear lipid droplets, and there was no inflammatory cell infiltration. However, liver tissue of the MOD group displayed observable pathological alterations, including a large number of round unstained lipid droplets in hepatocytes, significant swelling of hepatocytes, inflammatory infiltration of lymphocytes, hyperemia of central vein cells, etc. This was in accordance with the pathological liver characteristics of rats with T2DM [41]. In contrast, the number of cells in liver lesions in the MT group and the MT+KBP group decreased significantly, the arrangement tended to be orderly, the number of vacuoles decreased, and the phenomenon of lymphocyte inflammatory infiltration was considerably alleviated. This indicated that it had a good effect on improving and repairing liver injury caused by T2DM, which was consistent with the research rule of Gong et al. [42].

### 3.5. Effect of the MT–Kidney Bean Polyphenol Complex on SCFAs in the Intestinal Contents of Rats with T2DM

**Figure 6:**
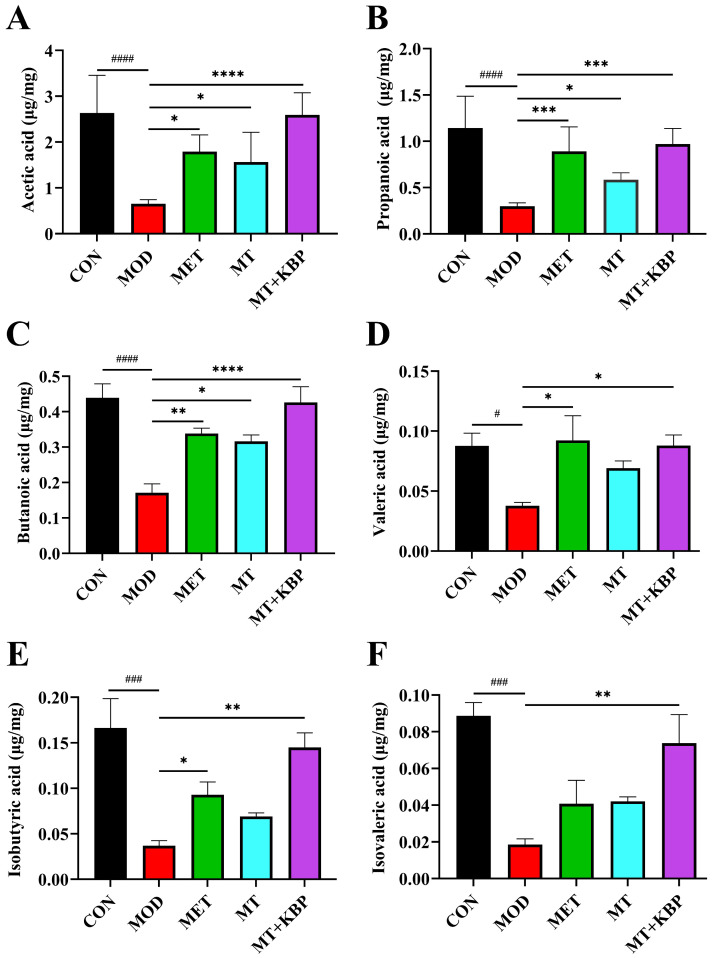
Effect of the MT–kidney bean polyphenol complex on SCFAs in intestinal contents of rats with T2DM. (**A**) Acetic acid; (**B**) Propanoic acid; (**C**) Butanoic acid; (**D**) Valeric acid; (**E**) Isobutyric acid; (**F**) Isovaleric acid. Data are expressed by mean ± standard deviation, *n* = 8. (# *p* < 0.05, ### *p* < 0.001, and #### *p* < 0.0001 versus CON; * *p* < 0.05, ** *p* < 0.01, *** *p* < 0.001, and **** *p* < 0.0001 versus MOD).

As the major metabolite of the intestinal flora, SCFAs can improve blood glucose homeostasis and insulin function [43]. In this study, the concentrations of six SCFAs in the intestinal contents of rats with T2DM were determined. The changes are shown in Figure 6. Compared to the CON group, the concentrations of acetic acid, propionic acid, butyric acid, valeric acid, isobutyric acid, and isovaleric acid in the MOD group decreased significantly (*p* < 0.05). After 7 weeks of intervention, the concentrations of six SCFAs in the intestinal contents of rats in the MET, MT, and MT+KBP groups were significantly higher than those in the MOD group (*p* < 0.05). Among them, the improvement effect of the MT+KBP group was the best, which increased by 296.03%, 223.86%, 148.97%, 132.63%, 292.58%, and 298.07%, respectively.

### 3.6. Effects of the MT–Kidney Bean Polyphenol Complex on the Diversity of the Gut Microbiota in Rats with T2DM

**Figure 7:**
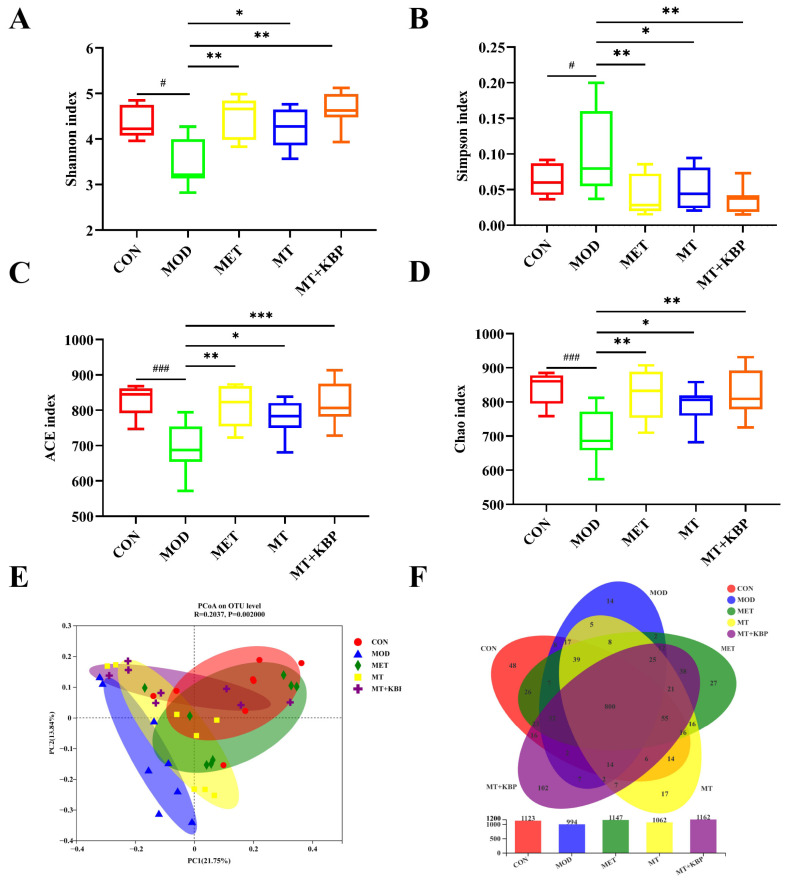
Effects of the MT–kidney bean polyphenol complex on the diversity of gut microbiota in rats with T2DM. (**A**) Shannon index of sample alpha diversity; (**B**) Simpson index of sample alpha diversity; (**C**) ACE index of sample alpha diversity; (**D**) Chao 1 index of sample alpha diversity; (**E**) Unweighted UniFrac PCoA; (**F**) Venn diagram. Data are expressed by mean ± standard deviation, *n* = 8. (# *p* < 0.05 and ### *p* < 0.001; versus CON; * *p* < 0.05, ** *p* < 0.01, and *** *p* < 0.001 versus MOD).

To explore the potential improvement associated with a high-fat diet combined with STZ-induced T2DM induced by the MT–kidney bean polyphenol complex, the gut microbiota diversity level, composition structure, and species differences in the intestinal contents of rats were analyzed using the 16S rRNA sequencing technology. Analysis of different α diversity indices can reflect the diversity and species richness of the gut microbiota in rats. The higher the Shannon, ACE, and Chao indices, the smaller the Simpson index, indicating that the greater the diversity of the gut microbiota, the richer the species. The Shannon index in the CON group was significantly higher than that in the MOD group (*p* < 0.05), while the Simpson index was significantly lower than that in the MOD group (*p* < 0.01), as shown in Figure 7A–D. Compared to the MOD group, the Shannon index increased considerably and the Simpson index decreased significantly in the MT and MT+KBP groups (*p* < 0.01). These results indicated that the MT–kidney bean polyphenol complex could increase the diversity of the gut microbiota in rats with T2DM. At the same time, ACE and Chao in the MET, MT, and MT+KBP groups were significantly higher than those in the MOD group. In addition, the index change in the MT+KBP group was highly significant (*p* < 0.01), which was the same as that in the MET group (*p* < 0.01). It can be seen that the MT–kidney bean polyphenol complex could regulate the richness of the intestinal microbiota. Furthermore, based on the principal coordinates analysis (PCoA), the differences and similarities of gut microbiota communities among different groups were reflected by β diversity. The distance was greater between the rats with T2DM in the MOD group and the intervention groups, indicating that different treatments have significant differences in the intestinal microbiota composition of diabetic rats (*p* < 0.001, R = 0.2037). The above intervention groups and the CON group samples developed along the same direction, indicating that the intestinal microflora of rats in each group was close to that of normal rats. Among them, the MET and MT+KBP groups were closer to the CON group on the whole, which indicates that the intestinal microflora of the experimental groups was more similar to that of normal rats. The number of common and unique OTUs in each experimental group was statistically analyzed using a Venn diagram. The diversity of intestinal microflora was reflected based on the unique OTU abundance.

As is shown in Figure 7E,F, the overlap and uniqueness of the OTU composition in different experimental groups can be seen. There were 800 OTUs shared among the groups, indicating an active core microbial community in the experimental group. In addition, the number of unique OTUs in the CON, MOD, MET, MT, and MT+KBP groups was 48, 14, 27, 17, and 102, respectively. The histograms showed that the MOD group had the minimum number of OTUs, and the MT+KBP group had the maximum number of OTUs, followed by the MET group. T2DM can reduce the intestinal microflora diversity of rats, and can be significantly improved by the MT–kidney bean polyphenol complex.

### 3.7. Effects of the MT–Kidney Bean Polyphenol Complex on the Composition of Gut Microbiota in Rats with T2DM

It has been proven that the functional components in kidney beans have a positive correlation in terms of the regulation of the composition of intestinal microflora, and they perform a particular function [44,45]. To further clarify the distribution of the MT–kidney bean polyphenol complex on the gut microbiota composition of rats with T2DM, the intestinal microflora composition was analyzed at the levels of phylum and genus.

**Figure 8:**
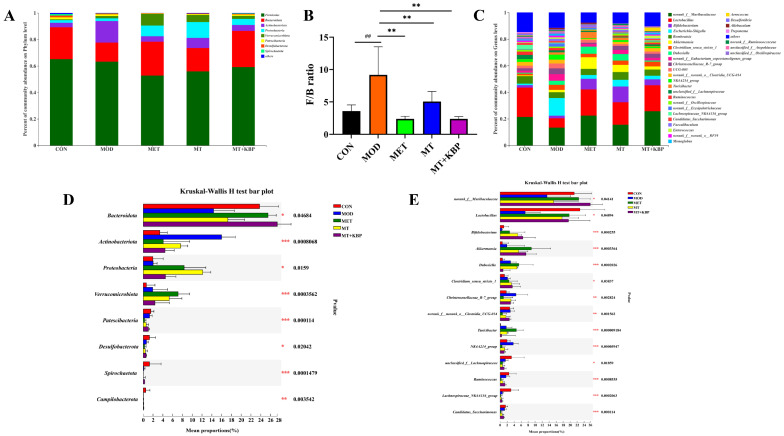
Effects of the MT–kidney bean polyphenol complex on the composition of gut microbiota in rats with T2DM. (**A**) Bacterial taxonomic profiling at the phylum level; (**B**) Ratio of Firmicutes and Bacteroidetes; (**C**) Bacterial taxonomic profiling at the genus level; (**D**) Statistical comparison of the relative abundance at the phylum level; (**E**) Statistical comparison of the relative abundance at the genus level. Data are expressed by mean ± standard deviation, *n* = 8. (## *p* < 0.01 versus CON; * *p* < 0.05, ** *p* < 0.01, and *** *p* < 0.001 versus MOD).

As is shown in Figure 8A, the dominant bacteria of the gut microbiota in each group at the phylum level were *Firmicutes*, *Bacteroidetes*, *Actinobacteria*, *Proteobacteria*, and *Verrucomicrobiota*, which was consistent with reported results [46,47]. The results showed that the abundance of *Bacteroidetes* decreased in rats with T2DM, and the change in *Bacteroidetes* abundance in the MOD group (from 24.05% to 14.37%) was in accordance with this research. In comparison to the MOD group, the abundance of *Bacteroidetes* in the MET, MT, and MT+KBP groups increased (25.53%, 17.63%, and 27.32%, respectively). Meanwhile, compared with the MOD group, the relative abundance of *Firmicutes* and *Actinobacteriota* decreased in the MET, MT, and MT+KBP groups, while *Proteobacteria* and *Verrucomicrobia* increased. This was consistent with the results of Wang et al. [48]. The ratio of *Firmicutes* to *Bacteroidetes* (F/B) can reflect the intestinal microflora disorder induced by a high-fat diet [49]. In this study, the F/B of the MOD group was the highest. In contrast, that of the MET and MT+KBP groups were the lowest (Figure 8B), which indicated that MT–kidney bean polyphenol complex could improve the intestinal microflora disorder induced by a high-fat diet in T2DM. At the genus level, the relative abundance of *norank_f_Muribaculaceae*, Lactobacillus, *Akkermansia*, and *Romboutsia* in the MET and MT+KBP groups were increased significantly, compared with the MOD group (Figure 8C). Of particular note were *Akkermansia* and *Romboutsia.* which have been reported to be related to T2DM [50]. Based on the Kruskal–Wallis H test bar plot, the microbial species with significant differences in different groups could be intuitively seen, as shown in Figure 8D,E. The *Bacteroidota*, *Proteobacteria*, and *Verrucomicrobiota* in the MET and MT+KBP groups differed significantly from those in the MOD group (*p* < 0.05, *p* < 0.01).

To further explore the specific differences between each sample, the LEfSe method was used to analyze biomarkers in the form of groups. Differences in microflora in the intestinal contents of rats were analyzed in the linear discriminant analysis score column (LDA) (LDA > 2, *p* < 0.05), as shown in Supplementary Figure S1A,B. The intestinal tract of rats in the CON group was significantly enriched with various beneficial intestinal bacteria, such as *Lactobacillus* and so on. However, *Escherichia–Shigella* was enriched in the MOD group. After the intervention, the MT+KBP group significantly enriched *Lactobacillales*, *Bacteroidales*, *p_Bacteroidota*, *Akkermansia*, *f_Muribaculaceae*, and *g_norank_f_Muribaculacea*, and decreased the abundance of *Escherichia–Shigella*.

Based on the results presented above, it could be found that the MT–kidney bean polyphenol complex has a certain impact on intestinal health. And, it also restored and reshaped the composition and structure of intestinal microflora in rats with T2DM by positively regulating the dominant differential bacteria in the intestinal tract, thus playing a role in interference with T2DM.

### 3.8. Correlation Analysis between the Biochemical Indicators and Gut Microbiota in Rats with T2DM 

**Figure 9:**
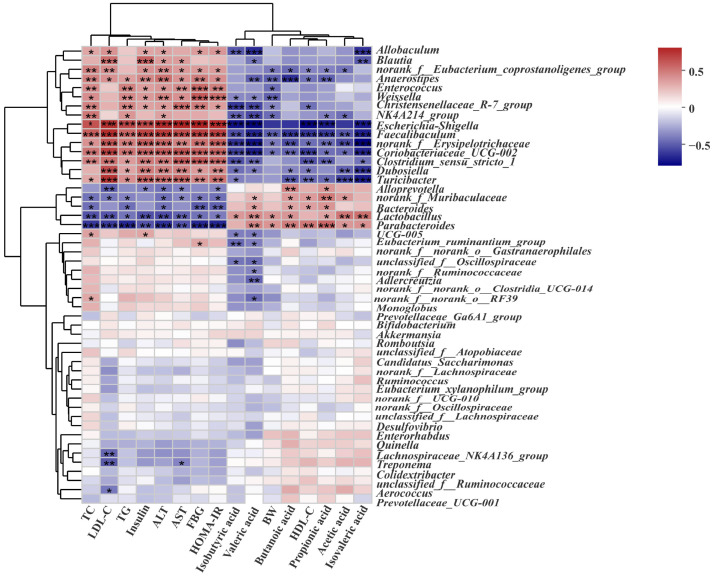
Correlation analysis between biochemical indicators and gut microbiota in rats with T2DM. Data are expressed by mean ± standard deviation, *n* = 8. (* *p* < 0.05, ** *p* < 0.01, and *** *p* < 0.001).

To explore the biochemical indicators related to intestinal bacteria and T2DM in different groups of samples, Spearman’s correlation analysis was used to reveal the potential correlation between intestinal microflora and apparent indicators. As is shown in Figure 9, different species of bacteria and apparent indicators showed a significant positive and negative correlation. For example, *Bacteroides* were negatively correlated with TG, ALT, and TC, but positively correlated with valeric acid, propionic acid, and HDL-C. *Alloprevotella*, *Allobaculum*, and *Blautia* were negatively associated with insulin, LDL-C, TG, ALT, AST, and HOMA-IR. The correlation between the abundance of intestinal microflora and the biochemical indicators mentioned above is consistent with some results of the study by Li et al. [31]. *Norank_f_Muribaculaceae*, *Parabacteroides*, and *Lactobacillus* were positively correlated with acetic acid and other SCFAs, but negatively correlated with TC and TG. The variation in intestinal microflora was the same as that of Ma et al. [51]. *Clostridium_sensu_stricto_1*, *Faecalibaculum*, *Coriobacteriaceae_UCG-002*, *Anaerostipes*, *Christensenellaceae_R-7_group*, and *norank_f_Erysipelotrichaceae* were negatively correlated with propionic acid and other SCFAs. However, these florae have a significant positive correlation with blood glucose and blood lipid indicators. *Escherichia–shigella*, *Weissella*, and *Enterococcus* were positively correlated with TC, TG, AST, ALT, insulin, FBG, and HOMA-IR. These bacteria belonged to harmful intestinal microorganisms and had some adverse effects on the blood-glucose-related indicators of T2DM.

## 4. Discussion

As a long-term metabolic disease, T2DM has become one of the top-ten factors leading to adult death [52]. At present, common antidiabetic drugs include α-glucosidase inhibitors, biguanides, sulfonylureas, and thiazolidinediones, but these drugs have specific side effects or toxicity [53]. In recent years, plant bioactive components have attracted significant attention due to their safe properties and good hypoglycemic effect [54]. In this study, the rat model of type 2 diabetes was induced by a high-fat diet and streptozotocin. The hypoglycemic effect of the MT–kidney bean polyphenol complex was systematically evaluated. The hypoglycemic mechanism was explored by analyzing the concentration of SCFAs, species abundance, diversity, and community composition of gut microbiota.

Weight loss is a typical feature of patients with T2DM [33]. In this study, the body weight of the rats in the MOD group was significantly lower than that in the other groups (Figure 3A), indicating that the rats in the model group had the typical symptoms of diabetes. However, the body weight of rats in each treatment group (MET, MT, and MT+KBP groups) increased significantly (*p* < 0.05).

A crucial indicator for the clinical diagnosis and tracking of diabetes mellitus development is FBG [55]. Similarly, the OGTT can easily demonstrate the ability of the body to regulate blood sugar and it is also widely used to diagnose prediabetes [56]. In this study, the FBG of the rats in the MOD group was (25.0 ± 0.56) mmol/L, which was much higher than the standard FBG of 11.1 mmol/L determined by diabetes, indicating that the model rats were in a state of severe hyperglycemia. After 7 weeks of intervention, the level of FBG in the MET, MT, and MT+KBP groups decreased significantly (*p* < 0.05). At the same time, the OGTT results showed that the glucose tolerance of the model rats was impaired (Figure 3C). Compared with the MOD group, the blood glucose level of the MT and MT+KBP groups decreased significantly during the entire period (*p* < 0.05). Insulin function is considered an essential factor in maintaining glucose homeostasis [57]. When blood glucose typically increases, insulin secretion will increase. However, because patients with T2DM have been in a state of hyperglycemia for a long time, insulin resistance is also one of the representative characteristics of T2DM [58]. The results of the levels of insulin in the MT and MT+KBP groups were significantly lower than those in the MOD group (*p* < 0.05). On the other hand, the HOMA-IR in the MT and MT+KBP groups was 31.53% and 45.34% lower than in the MOD group, respectively. As an important organ for secreting insulin, the pancreas plays a vital role in regulating blood glucose levels [59]. However, long-term exposure to a high-fat and high-glucose blood environment will lead to pancreatic tissue damage and the dysfunction of islet β-cells, subsequently inducing T2DM [60]. Histological sections of the pancreas in the current study showed that the MT–kidney bean polyphenol complex could effectively improve the inflammatory infiltration and proliferation of islet cells in rats with T2DM, resulting in the integrity of islet cell morphology and the recovery of pancreatic function (Figure 5). This result was consistent with the previous findings of HOMA-IR and was similar to the result of Zhang et al. [58]. These results regarding blood-glucose-related indicators and pancreatic tissue suggested that the MT–kidney bean polyphenol complex could effectively regulate blood glucose levels, restore insulin function, relieve the symptoms of insulin resistance, and repair pancreatic damage in rats with T2DM. And, the hypoglycemic effect of the MT–kidney bean polyphenol complex was higher than that of the single MT. This result may be related to the improvement in its bioavailability after the interaction, and is also consistent with the conclusions of Zhou et al. and Li et al. [38,61].

Excess glucose in the blood of patients with T2DM is constantly converted to fat, so abnormal fat metabolism often occurs [62]. In this experiment, the levels of TG, TC, and LDL-C in the MT and MT+KBP groups were significantly lower than those in the MOD group (*p* < 0.05), while HDL-C showed the opposite result (Figure 4A–D). Previous studies have shown that T2DM often leads to non-alcoholic fatty liver disease (NAFLD) [63]. As the two most important biochemical markers of liver function, ALT and AST have also been proven to indirectly reflect the status of T2DM [64]. In this study, the levels of ALT and AST in the MET, MT, and MT+KBP groups were significantly lower than those in the MOD group (*p* < 0.05), but there was no significant difference between MT and the MT–kidney bean polyphenol complex on the levels of ALT and AST (Figure 4E,F). Furthermore, as one of the critical insulin-sensitive tissues, liver status is closely related to T2DM [65]. The results of this study demonstrated that the MT–kidney bean polyphenol complex could alleviate pathological conditions such as hepatocyte swelling, inflammatory infiltration, and congestion in the liver of rats with T2DM (Figure 5). This result was consistent with the results of ALT and AST, and was also compatible with the study by Gong et al. [42]. These results indicated that the MT–kidney bean polyphenol complex could improve blood lipid levels and repair liver damage in rats with T2DM.

SCFA is a type of microbial metabolite that can promote fat and carbohydrate metabolism [66]. Zhao et al. found that a reduction in SCFA production may be one of the factors that leads to the occurrence of T2DM [13]. The three main SCFAs, including acetic acid, propionic acid, and butyric acid, have been reported to account for more than 95% of SCFAs [67]. They play an important role in glucose and lipid metabolism [68]. In addition, acetic acid and propionic acid have positive effects on regulating body weight and insulin sensitivity [69]. Butyric acid has the function of maintaining the stability of the intestinal environment and preventing colon cancer [70]. In the present study, SCFA concentrations in the intestinal contents of five groups of rats were determined (Figure 6). The results indicated that the MT–kidney bean polyphenol complex could achieve its anti-diabetic effect by promoting SCFA production in the intestinal tract of rats with T2DM.

Several studies have proven that intestinal microflora dysbiosis will lead to metabolic disorders, and then increase the risk of T2DM and other metabolic diseases [71,72]. Therefore, researchers continue to focus on the relationship between T2DM and gut microbiota, to reveal whether functional components in the diet can achieve the purpose of T2DM intervention by improving the gut microbiota. The results of previous studies have confirmed that the α-diversity of the gut microbiota is generally decreased in the gut of diabetic patients, while the β-diversity shows differences [73]. The results of this study were also consistent with this view. The richness and structural diversity of gut microbiota in the MOD group induced by a high-fat diet were low. Moreover, the diversity and species richness of the gut microbiota were improved after the intervention of the MT–kidney bean polyphenol complex.

The increase in the *Firmicutes*/*Bacteroidetes* (F/B) ratio is an important marker of intestinal microflora imbalance in T2DM, which is negatively correlated with glucose homeostasis and insulin sensitivity [74]. In this study, in the MOD group, *Firmicutes* abundance was higher, *Bacteroidetes* abundance was the lowest, and F/B was higher. This was consistent with the results reported above. As expected, the abundance of *Bacteroidetes* in rats with T2DM was increased, and the F/B value was significantly decreased (*p* < 0.01) after the intervention of MT and MT–kidney bean polyphenol complex. At the same time, the abundance of *Bacteroidetes* in the MT+KBP group was significantly higher than that in the MT group (*p* < 0.05). And, the blood-glucose-related indicators in the early stage were also better than those in the MT group, indicating that the effect of the MT–kidney bean polyphenol complex on T2DM was better than that of MT. *Verrucomicrobiota* and *Patescibacteria* have been reported to potentially improve T2DM [75]. In this study, the abundance of these two genera increased, which indicated that the intervention of the MT–kidney bean polyphenol complex had an obvious positive regulation effect on related bacteria in T2DM. Previous researchers have found that the abundance of *Akkermansia* was negatively correlated with blood glucose and inflammatory factors at the genus level, which could be considered a potential biomarker of intestinal health in the treatment of T2DM [76]. On top of this, some studies have proven that dietary polyphenols can maintain the balance of intestinal microorganisms by promoting an increased abundance of *Akkermansia* in the intestinal tract [77,78]. Therefore, we speculate that the MT–kidney bean polyphenol complex could maintain intestinal health by stimulating the growth and colonization of anti-inflammatory microbial communities (such as *Akkermansia*). *Lachnospiraceae_NK4A136_group* is a potentially beneficial bacterium for preventing obesity and T2DM [79]. In this experiment, the abundance of *Lachnospiraceae_NK4A136_group* was higher in the CON group and significantly higher in the intervention group than in the MOD group. However, other studies have shown that this genus is positively correlated with the development of T2DM, and the difference in results requires further studies on the correlation between this genus and T2DM [58]. *Muribaculaceae* is believed to have a negative relationship with metabolic syndromes, which are caused by a high-fat diet and include obesity, inflammatory bowel disease, and diabetes [80]. In the results of this experiment, the abundance increased after intervention, further indicating that the MT–kidney bean polyphenol complex could prevent or improve metabolic syndrome by regulating the gut microbiota of T2DM. These results may be due to the synergistic effect that may enhance the function of MT when it binds to kidney bean polyphenol to form complexes. Through an extensive study of human intestinal microorganisms, it has been found that butyric-acid-producing bacteria are mainly distributed in *Clostridium* [81]. In this study, it was found that the abundance of *Clostridium sensu stricto 1* was higher in the MT and MT+KBP groups. And, the content of butyric acid production was also higher than that in the MOD group, which may mean that the MT–kidney bean polyphenol complex could promote the enrichment and metabolism of *Clostridium* to produce butyric acid and play a positive protective role in intestinal health.

Increasing evidence indicates that the mechanism of dietary intervention in metabolic diseases induced by a high-fat diet is strongly related to the regulation of the gut microbiota [48]. Therefore, it is necessary to explore the potential relationship between gut microbiota with high intestinal abundance and apparent indices and blood indices by correlation analysis. It has been reported that *Bacteroidetes* can promote the enrichment of acetic acid and propionic acid [82]. In this study, *Bacteroidetes* showed a positive correlation with the above two SCFAs, and a positive correlation with butyric acid and valeric acid in different degrees. At the same time, *Bacteroidetes* showed a negative correlation with blood lipid and blood glucose indices (Figure 7). These results indicated that this genus was involved in the metabolism of glucose and lipid in T2DM, which also coincides with the existing literature [83]. The imbalance between probiotics and pathogenic bacteria leads to an imbalance in gut microbiota, and changes in the type and content of microbial metabolites. This leads to chronic inflammation, which has been identified as one of the key risk factors for the development of diabetes. It has been reported that the abundances of *Blautia*, *Bifidobacterium*, *Alloprevotella*, and *Clostridium* were down-regulated in the intestinal tract of diabetic patients [84]. At the same time, the bacteria mentioned above have also been proven to be SCFAs-producing bacteria, which play an active role in alleviating inflammation [85]. In the intervention groups of this experiment, as mentioned above, the abundance of bacteria increased, and the content of SCFAs also showed an increasing trend. There was a positive correlation between them, and the former was negatively correlated with the blood indices of rats with T2DM. The metabolites of different intestinal flora are different, making the functions of various metabolites different. The reason for this may be that the enriched probiotics inhibit pathogenic bacteria and promote intestinal health. The obvious improvement in the intestinal tract of T2DM induced by the MT–kidney bean polyphenol complex may be related to the enrichment of these beneficial bacteria.

In this study, *Allobaculum* was not only negatively correlated with LDL-C, TC, and HOMA-IR, but also positively correlated with butyrate. This result may be explained by the findings of Obanda et al. [86]. They found that *Allobaculum* plays a variety of roles in regulating inflammation and lipid metabolism, reducing colonic inflammation by producing 3-hydroxy octadecadienoic acid, and it is accompanied by a significant reduction in the LDL-C level. *Escherichia–shigella* can promote inflammation, intestinal barrier damage, and glucose metabolism disorder [87]. From our results, *Escherichia–shigella* was detected in the MOD group and showed a positive link with *Escherichia–shigella* and the indices of blood glucose and blood lipid in rats. This indicated that *Escherichia–shigella* increased the probability of intestinal pathogenesis in T2DM. This result may be the key factor leading to abnormal glucose and lipid metabolism. This result can provide a reference for future research on dietary functional components through the targeted regulation of bacteria.

It has been found that casein, whey protein isolate, and bovine serum albumin can improve the stability and bioavailability of polyphenols through non-covalent binding (hydrogen bonds, hydrophobic interaction, and ion interaction) [88,89]. In this study, we observed that, compared with MT alone, the MT–kidney bean polyphenol complex has a better effect on improving insulin resistance and regulating the gut microbiota in rats with T2DM. This result is also consistent with the findings of many researchers. For example, Ribicky et al. found that the bioavailability of tarragon polyphenols in C57BL/6 mice increased after interacting with soy protein isolate (SPI), which was attributed to the stabilizing effect of soy protein [90]. Li et al. also found that the hypoglycemic effect of young apple polyphenols (YAP) and whey protein isolate (WPI) was 6.85% higher than that of YAP alone. This was due to the synergistic effect of YAP and WPI, which enhanced the functional effect of polyphenols [61]. Polyphenols have high reactivity and are easily oxidized into corresponding quinones, which can react covalently with nucleophiles, such as free amino groups, lysine, cysteine, and tryptophan of proteins [91]. Metallothionein is rich in cysteine (30–33%) [17], so it can interact with kidney bean polyphenols, thus delaying the enzymatic hydrolysis of kidney bean polyphenols during digestion and improving the bioavailability of kidney bean polyphenols. Meanwhile, the existence of phenolic compounds may also lead to the partial unfolding of protein structures, thus increasing the accessibility of sensitive peptide bonds [92,93]. To sum up, in this study, metallothionein encapsulates and protects kidney bean polyphenols in its unique spatial structure by non-covalent binding (Figure 2B), which results in the MT–kidney bean polyphenol complex formed by metallothionein having a better hypoglycemic effect.

## 5. Conclusions

To conclude, this study explained the hypoglycemic effect of the MT–kidney bean polyphenol complex on rats with T2DM. The MT–kidney bean polyphenol complex can regulate blood glucose and blood lipid levels in rats with T2DM, alleviate insulin resistance, and alleviate the damage to the pancreas and liver tissue caused by T2DM. In addition, the MT–kidney bean polyphenol complex can also increase the concentration of SCFAs in the intestines of rats, regulate the diversity and composition of gut microbiota, and restore the biological balance of intestinal microflora, thus having a positive intervention effect on rats with T2DM. All of these results show the potential of MT–kidney bean polyphenol complex as a natural component in treating diabetes. At the same time, this study also provides a theoretical basis for the interaction between dietary polyphenols and proteins in the application of metabolic diseases. In subsequent studies, we will explore how the MT–kidney bean polyphenol complex regulates the blood glucose signaling pathway to further elucidate its hypoglycemic mechanism.

## Data Availability

The data presented in this study are available on request from the corresponding author.

## Supplementary Materials

The following supporting information can be downloaded at: https://www.mdpi.com/article/10.3390/foods12163139/s1, Figure S1: Effects of MT–kidney bean polyphenol complex on the composition of gut microbiota in rats with T2DM. (A) Indicator bacteria of gut microbiota with LDA scores in rats (LDA > 2, *p* < 0.05); (B) LEfSe cladogram; Table S1: Compositions and contents of polyphenolic compounds in the purified kidney bean polyphenol extracts.
