# Supplementary material for: Metallothionein–Kidney Bean Polyphenol Complexes Showed Antidiabetic Activity in Type 2 Diabetic Rats by Improving Insulin Resistance and Regulating Gut Microbiota
Source: Foods. 2023 Aug 21;12(16):3139. doi: 10.3390/foods12163139 (PMC10453533; doi:10.3390/foods12163139)
Supplement: Supplementary file 1 [file foods-12-03139-s001.zip › Supplementary figure S1.pdf]

Supplementary figure S1. Effects of MT–kidney bean polyphenol complex on the composition of gut microbiota in rats with T2DM. (A) Indicator bacteria of gut microbiota with LDA scores in rats (LDA > 2, $p < 0.05$); (B) LEfSe cladogram.

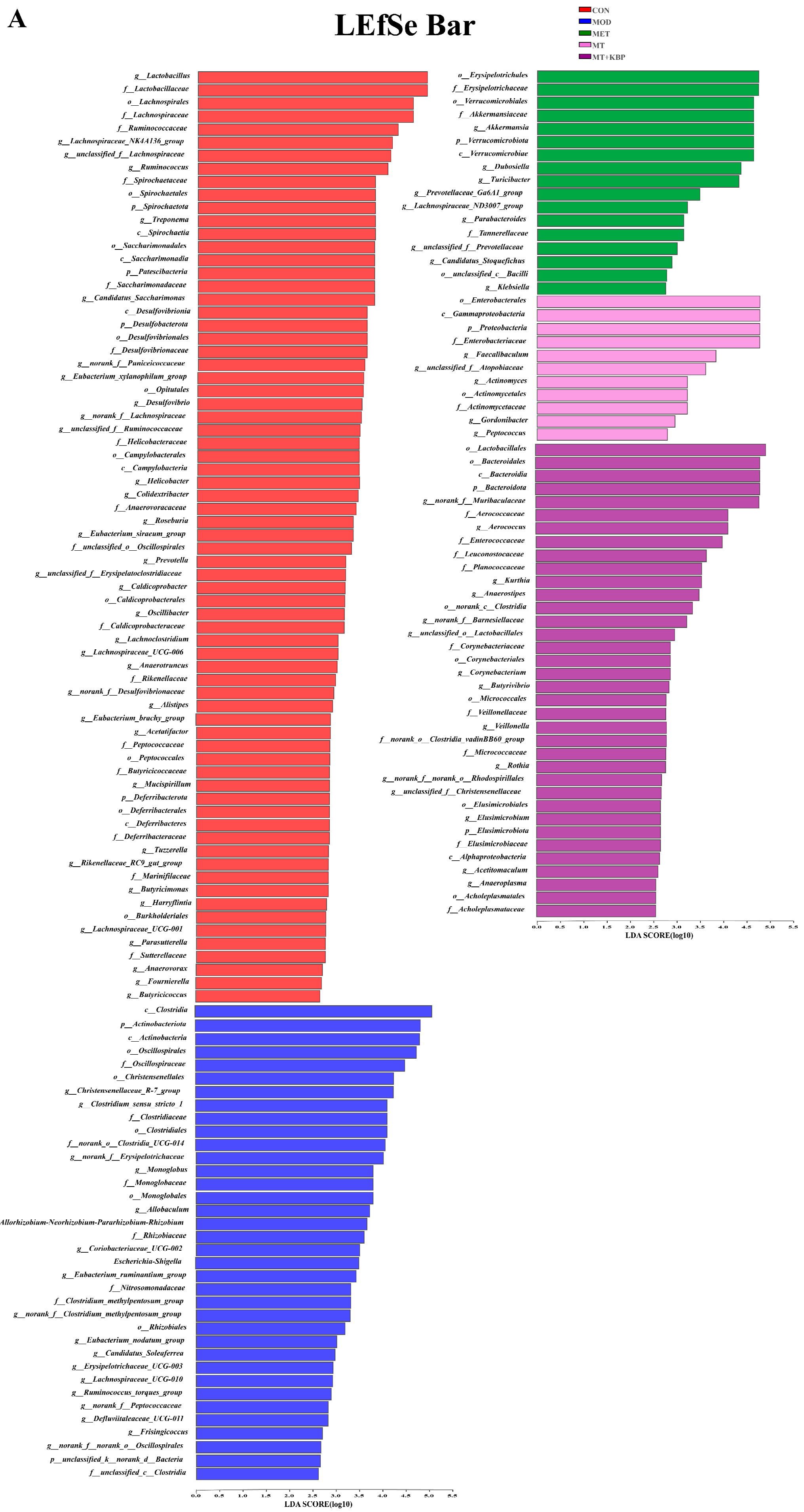
A
LEfSe Bar
CON
MOD
MET
MT
MT+KBP
g__Lactobacillus
f__Lactobacillaceae
o__Lachnospirales
f__Lachnospiraceae
f__Ruminococcaceae
g__Lachnospiraceae_NK4A136_group
g__unclassified_f__Lachnospiraceae
g__Ruminococcus
f__Spirochaetaceae
o__Spirochaetales
p__Spirochaetota
g__Treponema
c__Spirochaetia
o__Saccharimonadales
c__Saccharimonadia
p__Patescibacteria
f__Saccharimonadaceae
g__Candidatus_Saccharimonas
c__Desulfovibrionia
p__Desulfobacterota
o__Desulfovibrionales
f__Desulfovibrionaceae
g__norank_f__Puniceicoccaceae
g__Eubacterium_xylanophilum_group
o__Opitutales
g__Desulfovibrio
g__norank_f__Lachnospiraceae
g__unclassified_f__Ruminococcaceae
f__Helicobacteraceae
o__Campylobacterales
c__Campylobacteria
g__Helicobacter
g__Colidextribacter
f__Anaerovoracaceae
g__Roseburia
g__Eubacterium_siraeum_group
f__unclassified_o__Oscillospirales
g__Prevotella
g__unclassified_f__Erysipelatoclostridiaceae
g__Caldicoprobacter
o__Caldicoprobacterales
g__Oscillibacter
f__Caldicoprobacteraceae
g__Lachnoclostridium
g__Lachnospiraceae_UCG-006
g__Anaerotruncus
f__Rikenellaceae
g__norank_f__Desulfovibrionaceae
g__Alistipes
g__Eubacterium_brachy_group
g__Acetatifactor
f__Peptococcaceae
o__Peptococcales
f__Butyricicoccaceae
g__Mucispirillum
p__Deferribacterota
o__Deferribacterales
c__Deferribacteres
f__Deferribacteraceae
g__Tuzzerella
g__Rikenellaceae_RC9_gut_group
f__Marinifilaceae
g__Butyricimonas
g__Harryflintia
o__Burkholderiales
g__Lachnospiraceae_UCG-001
g__Parasutterella
f__Sutterellaceae
g__Anaerovorax
g__Fournierella
g__Butyricicoccus
c__Clostridia
p__Actinobacteriota
c__Actinobacteria
o__Oscillospirales
f__Oscillospiraceae
o__Christensenellales
g__Christensenellaceae_R-7_group
g__Clostridium_sensu_stricto_1
f__Clostridiaceae
o__Clostridiales
f__norank_o__Clostridia_UCG-014
g__norank_f__Erysipelotrichaceae
g__Monoglobus
f__Monoglobaceae
o__Monoglobales
g__Allobaculum
g__Allorhizobium-Neorhizobium-Pararhizobium-Rhizobium
f__Rhizobiaceae
g__Coriobacteriaceae_UCG-002
Escherichia-Shigella
g__Eubacterium_ruminantium_group
f__Nitrosomonadaceae
f__Clostridium_methylpentosum_group
g__norank_f__Clostridium_methylpentosum_group
o__Rhizobiales
g__Eubacterium_nodatum_group
g__Candidatus_Soleaferrea
g__Erysipelotrichaceae_UCG-003
g__Lachnospiraceae_UCG-010
g__Ruminococcus_torques_group
g__norank_f__Peptococcaceae
g__Defluviitaleaceae_UCG-011
g__Frisingicoccus
g__norank_f__norank_o__Oscillospirales
p__unclassified_k__norank_d__Bacteria
f__unclassified_c__Clostridia
0.0 0.5 1.0 1.5 2.0 2.5 3.0 3.5 4.0 4.5 5.0 5.5
LDA SCORE(log10)
o__Erysipelotrichales
f__Erysipelotrichaceae
o__Verrucomicrobiales
f__Akkermansiaceae
g__Akkermansia
p__Verrucomicrobiota
c__Verrucomicrobiae
g__Dubosiella
g__Turicibacter
g__Prevotellaceae_Ga6A1_group
g__Lachnospiraceae_ND3007_group
g__Parabacteroides
f__Tannerellaceae
g__unclassified_f__Prevotellaceae
g__Candidatus_Stoquefichus
o__unclassified_c__Bacilli
g__Klebsiella
o__Enterobacterales
c__Gammaproteobacteria
p__Proteobacteria
f__Enterobacteriaceae
g__Faecalibaculum
g__unclassified_f__Atopobiaceae
g__Actinomyces
o__Actinomycetales
f__Actinomycetaceae
g__Gordonibacter
g__Peptococcus
o__Lactobacillales
o__Bacteroidales
c__Bacteroidia
p__Bacteroidota
g__norank_f__Muribaculaceae
f__Aerococcaceae
g__Aerococcus
f__Enterococcaceae
f__Leuconostocaceae
f__Planococcaceae
g__Kurthia
g__Anaerostipes
o__norank_c__Clostridia
g__norank_f__Barnesiellaceae
g__unclassified_o__Lactobacillales
f__Corynebacteriaceae
o__Corynebacteriales
g__Corynebacterium
g__Butyrivibrio
o__Micrococcales
f__Veillonellaceae
g__Veillonella
f__norank_o__Clostridia_vadinBB60_group
f__Micrococcaceae
g__Rothia
g__norank_f__norank_o__Rhodospirillales
g__unclassified_f__Christensenellaceae
o__Elusimicrobiales
g__Elusimicrobium
p__Elusimicrobiota
f__Elusimicrobiaceae
c__Alphaproteobacteria
g__Acetitomaculum
g__Anaeroplasma
o__Acholeplasmatales
f__Acholeplasmataceae
0.0 0.5 1.0 1.5 2.0 2.5 3.0 3.5 4.0 4.5 5.0 5.5
LDA SCORE(log10)

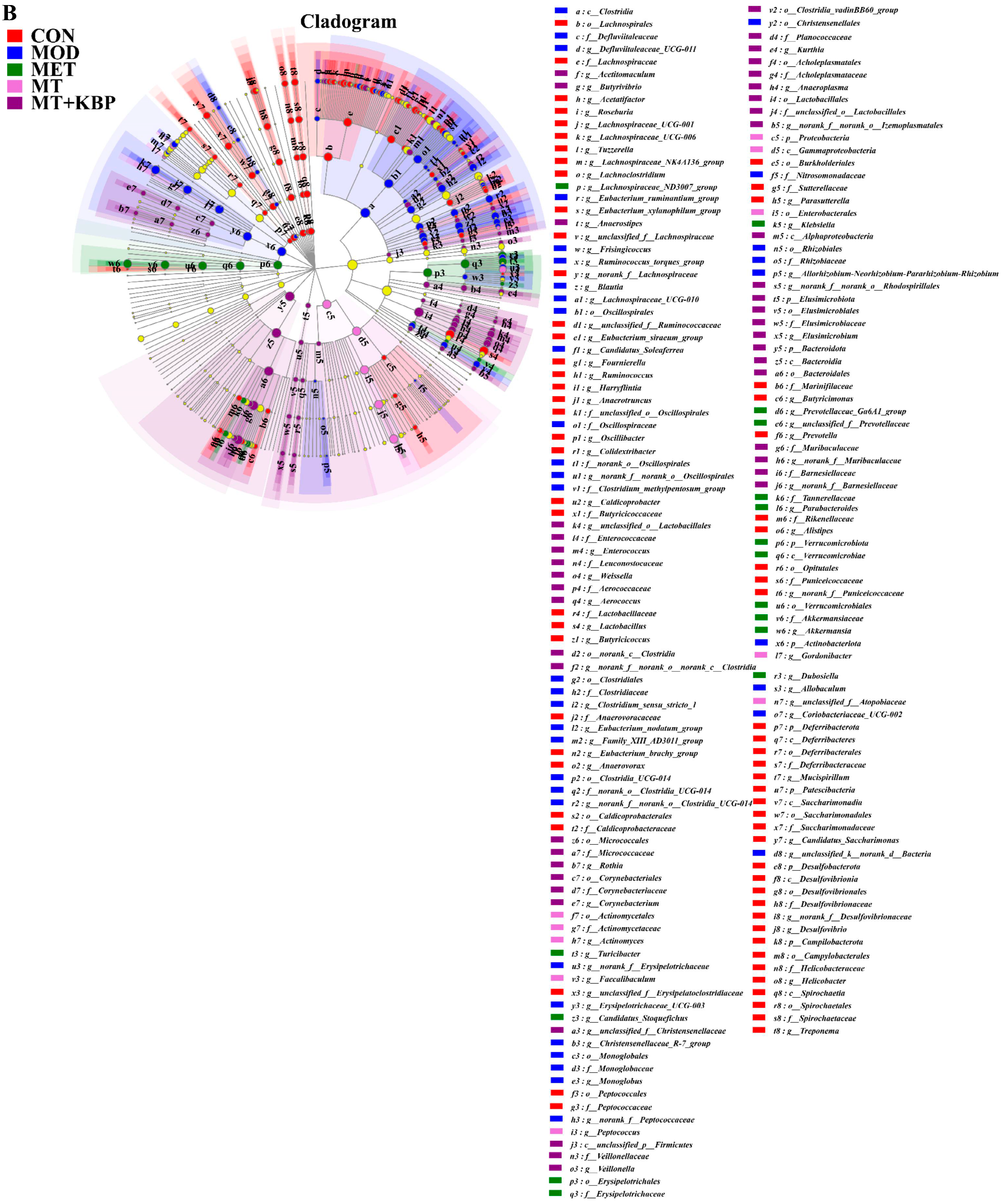
B
CON
MOD
MET
MT
MT+KBP
Cladogram
a : c__Clostridia
b : o__Lachnospirales
c : f__Defluviitaleaceae
d : g__Defluviitaleaceae_UCG-011
e : f__Lachnospiraceae
f : g__Acetitomaculum
g : g__Butyrivibrio
h : g__Acetatifactor
i : g__Roseburia
j : g__Lachnospiraceae_UCG-001
k : g__Lachnospiraceae_UCG-006
l : g__Tuzzerella
m : g__Lachnospiraceae_NK4A136_group
o : g__Lachnoclostridium
p : g__Lachnospiraceae_ND3007_group
r : g__Eubacterium_ruminantium_group
s : g__Eubacterium_xylanophilum_group
t : g__Anaerostipes
v : g__unclassified_f__Lachnospiraceae
w : g__Frisingicoccus
x : g__Ruminococcus_torques_group
y : g__norank_f__Lachnospiraceae
z : g__Blautia
a1 : g__Lachnospiraceae_UCG-010
b1 : o__Oscillospirales
d1 : g__unclassified_f__Ruminococcaceae
e1 : g__Eubacterium_siraeum_group
f1 : g__Candidatus_Soleaferrea
g1 : g__Fournierella
h1 : g__Ruminococcus
i1 : g__Harryflintia
j1 : g__Anaerotruncus
k1 : f__unclassified_o__Oscillospirales
o1 : f__Oscillospiraceae
p1 : g__Oscillibacter
r1 : g__Colidextribacter
t1 : f__norank_o__Oscillospirales
u1 : g__norank_f__norank_o__Oscillospirales
v1 : f__Clostridium_methylpentosum_group
u2 : g__Caldicoprobacter
x1 : f__Butyricicoccaceae
k4 : g__unclassified_o__Lactobacillales
l4 : f__Enterococcaceae
m4 : g__Enterococcus
n4 : f__Leuconostocaceae
o4 : g__Weissella
p4 : f__Aerococcaceae
q4 : g__Aerococcus
r4 : f__Lactobacillaceae
s4 : g__Lactobacillus
z1 : g__Butyricicoccus
d2 : o__norank_c__Clostridia
f2 : g__norank_f__norank_o__norank_c__Clostridia
g2 : o__Clostridiales
h2 : f__Clostridiaceae
i2 : g__Clostridium_sensu_stricto_1
j2 : f__Anaerovoracaceae
l2 : g__Eubacterium_nodatum_group
m2 : g__Family_XIII_AD3011_group
n2 : g__Eubacterium_brachy_group
o2 : g__Anaerovorax
p2 : o__Clostridia_UCG-014
q2 : f__norank_o__Clostridia_UCG-014
r2 : g__norank_f__norank_o__Clostridia_UCG-014
s2 : o__Caldicoprobacterales
t2 : f__Caldicoprobacteraceae
z6 : o__Micrococcales
a7 : f__Micrococcaceae
b7 : g__Rothia
c7 : o__Corynebacteriales
d7 : f__Corynebacteriaceae
e7 : g__Corynebacterium
f7 : o__Actinomycetales
g7 : f__Actinomycetaceae
h7 : g__Actinomyces
t3 : g__Turicibacter
u3 : g__norank_f__Erysipelotrichaceae
v3 : g__Faecalibaculum
x3 : g__unclassified_f__Erysipelatoclostridiaceae
y3 : g__Erysipelotrichaceae_UCG-003
z3 : g__Candidatus_Stoquefichus
a3 : g__unclassified_f__Christensenellaceae
b3 : g__Christensenellaceae_R-7_group
c3 : o__Monoglobales
d3 : f__Monoglobaceae
e3 : g__Monoglobus
f3 : o__Peptococcales
g3 : f__Peptococcaceae
h3 : g__norank_f__Peptococcaceae
i3 : g__Peptococcus
j3 : c__unclassified_p__Firmicutes
n3 : f__Veillonellaceae
o3 : g__Veillonella
p3 : o__Erysipelotrichales
q3 : f__Erysipelotrichaceae
v2 : o__Clostridia_vadinBB60_group
y2 : o__Christensenellales
d4 : f__Planococcaceae
e4 : g__Kurthia
f4 : o__Acholeplasmatales
g4 : f__Acholeplasmataceae
h4 : g__Anaeroplasma
i4 : o__Lactobacillales
j4 : f__unclassified_o__Lactobacillales
b5 : g__norank_f__norank_o__Izemoplasmatales
c5 : p__Proteobacteria
d5 : c__Gammaproteobacteria
e5 : o__Burkholderiales
f5 : f__Nitrosomonadaceae
g5 : f__Sutterellaceae
h5 : g__Parasutterella
i5 : o__Enterobacterales
k5 : g__Klebsiella
m5 : c__Alphaproteobacteria
n5 : o__Rhizobiales
o5 : f__Rhizobiaceae
p5 : g__Allorhizobium-Neorhizobium-Pararhizobium-Rhizobium
s5 : g__norank_f__norank_o__Rhodospirillales
t5 : p__Elusimicrobiota
v5 : o__Elusimicrobiales
w5 : f__Elusimicrobiaceae
x5 : g__Elusimicrobium
y5 : p__Bacteroidota
z5 : c__Bacteroidia
a6 : o__Bacteroidales
b6 : f__Marinifilaceae
c6 : g__Butyricimonas
d6 : g__Prevotellaceae_Ga6A1_group
e6 : g__unclassified_f__Prevotellaceae
f6 : g__Prevotella
g6 : f__Muribaculaceae
h6 : g__norank_f__Muribaculaceae
i6 : f__Barnesiellaceae
j6 : g__norank_f__Barnesiellaceae
k6 : f__Tannerellaceae
l6 : g__Parabacteroides
m6 : f__Rikenellaceae
o6 : g__Alistipes
p6 : p__Verrucomicrobiota
q6 : c__Verrucomicrobiae
r6 : o__Opitutales
s6 : f__Puniceicoccaceae
t6 : g__norank_f__Puniceicoccaceae
u6 : o__Verrucomicrobiales
v6 : f__Akkermansiaceae
w6 : g__Akkermansia
x6 : p__Actinobacteriota
l7 : g__Gordonibacter
r3 : g__Dubosiella
s3 : g__Allobaculum
n7 : g__unclassified_f__Atopobiaceae
o7 : g__Coriobacteriaceae_UCG-002
p7 : p__Deferribacterota
q7 : c__Deferribacteres
r7 : o__Deferribacterales
s7 : f__Deferribacteraceae
t7 : g__Mucispirillum
u7 : p__Patescibacteria
v7 : c__Saccharimonadia
w7 : o__Saccharimonadales
x7 : f__Saccharimonadaceae
y7 : g__Candidatus_Saccharimonas
d8 : g__unclassified_k__norank_d__Bacteria
e8 : p__Desulfobacterota
f8 : c__Desulfovibrionia
g8 : o__Desulfovibrionales
h8 : f__Desulfovibrionaceae
i8 : g__norank_f__Desulfovibrionaceae
j8 : g__Desulfovibrio
k8 : p__Campilobacterota
m8 : o__Campylobacterales
n8 : f__Helicobacteraceae
o8 : g__Helicobacter
q8 : c__Spirochaetia
r8 : o__Spirochaetales
s8 : f__Spirochaetaceae
t8 : g__Treponema
