# Supplementary material for: Metallothionein–Kidney Bean Polyphenol Complexes Showed Antidiabetic Activity in Type 2 Diabetic Rats by Improving Insulin Resistance and Regulating Gut Microbiota
Source: Foods. 2023 Aug 21;12(16):3139. doi: 10.3390/foods12163139 (PMC10453533; doi:10.3390/foods12163139)
Supplement: Supplementary file 1 [file foods-12-03139-s001.zip › Supplementary table S1.pdf]

Supplementary table S1. Compositions and contents of polyphenolic compounds in the purified kidney bean polyphenol extracts.

Compositions	Contents (ng/mL)
Gallic acid	8.446 ± 0.032
Phenylalanine	264.288 ± 0.932
3,4-Dihydroxybenzoic acid	1547.064 ± 15.085
Protocatechualdehyde	656.613 ± 4.648
4-Hydroxybenzoic acid	248.233 ± 6.433
Catechin	699.897 ± 7.216
Vanillic acid	329.067 ± 2.268
Caffeic acid	87.172 ± 1.356
Syringic acid	78.881 ± 0.905
L-Epicatechin	530.920 ± 10.383
Vanillin	79.399 ± 1.443
p-Hydroxycinnamic Acid	520.272 ± 2.799
Syringaldehyde	3.397 ± 0.067
Salicylic acid	57.978 ± 0.630
Ferulic acid	2094.553 ± 16.945
Sinapic Acid	83.454 ± 0.209
Benzoic acid	524.783 ± 4.789
Hydrocinnamic acid	1.579 ± 0.041
Trans-Cinnamic acid	476.874 ± 6.710
